# Transcriptome and metabolome analyses of lignin biosynthesis mechanism of* Platycladus orientalis*

**DOI:** 10.7717/peerj.14172

**Published:** 2022-11-02

**Authors:** Ying Li, Qikui Wu, Xiaoyan Men, Fusheng Wu, Qian Zhang, Weinan Li, Limin Sun, Shiyan Xing

**Affiliations:** 1 State Forestry and Grassland Administration Key Laboratory of Silviculture in Downstream Areas of the Yellow River, Forestry College of Shandong Agricultural University, Taian, Shandong, China; 2 Shandong Forest and Grass Germplasm Resources Center, Jinan, Shandong, China; 3 Shandong Academy of Forestry Sciences, Jinan, Shandong, China

**Keywords:** *P. orientalis*, Lignin, Transcriptome, Metabolome, Synthesis mechanism

## Abstract

**Background:**

*Platycladus orientalis*, as an important plant for ecological protection, is a pioneer tree species for afforestation in arid and barren mountainous areas. Lignin has the functions of water and soil conservation, strengthening plant mechanical strength and resisting adverse environmental effects and plays an important role in the ecological protection benefits of *P. orientalis*.

**Methods:**

In this study, annual dynamic observations of the lignin content in roots, stems and leaves of one-year-old seedlings of a *P. orientalis* half-sib family were carried out, and combined transcriptome and metabolome analyses were carried out during three key stages of *P. orientalis* stem development.

**Results:**

The lignin contents in roots, stems and leaves of *P. orientalis* showed extremely significant spatiotemporal differences. In the stems, lignin was mainly distributed in the cell walls of the pith, xylem, phloem, pericyte, and epidermis, with differences in different periods. A total of 226 metabolites were detected in the stem of *P. orientalis*, which were divided into seven categories, including 10 synthetic precursor compounds containing lignin. Among them, the content of coniferyl alcohol was the highest, accounting for 12.27% of the total content, and caffeyl alcohol was the lowest, accounting for 7.05% only. By annotating the KEGG functions, a large number of differentially expressed genes and differential metabolites were obtained for the comparison combinations, and seven key enzymes and 24 related genes involved in the process of lignin synthesis in *P. orientalis* were selected.

**Conclusions:**

Based on the results of the metabolic mechanism of lignin in *P. orientalis* by biochemical, anatomical and molecular biological analyzes, the key regulatory pathways of lignin in *P. orientalis* were identified, which will be of great significance for regulating the lignin content of *P. orientalis* and improving the adaptability and resistance of this plant.

## Introduction

*Platycladus orientalis* (L.) Franco is a plant belonging to the Cupressaceae family. It is a traditional tree species that is native to China (Hu et al., 2019). It has strong climate adaptability and resistance to drought and barrenness. *P. orientalis* is a pioneer tree species for the greening of barren hills in North China, Northwest China, East China and northern South China (Zhang et al., 2016), and its seeds, timber, leaves, bark and roots are important raw materials for many domestic and industrial products. This species has received a lot of attention because of its great medicinal, ornamental, and ecological value, and is widely used across China. Lignin is a secondary metabolite that is second only to cellulose in terms of plant content. It plays an important biological role in the growth and development of plants (Rogers et al., 2005; Marie et al., 1998) and is mainly involved in enhancing the mechanical strength of plants, diverting the transport of water and nutrients, preventing the transport of pathogens, and enhancing the defense against various stresses (Ride & Barber, 1987; Zhao & Dixon, 2011).

With phenylalanine as a precursor, the process of lignin biosynthesis can be divided into three stages: phenylpropanoid metabolism, monolignol synthesis, and lignin polymerization (Vanholme et al., 2010). First, phenylalanine is deaminated by phenylalanine ammonia-lyase (PAL), and then catalyzed to form coumarin-CoA by cinnamate 4-hydroxylase (C4H) and 4-hydroxycinnamate CoA ligase (4CL) enzymes (Vogt, 2010). A number of enzymes play a key role in lignin monomer formation by coumarin-CoA (Dixon & Barros, 2019), including *p*-coumarate 3-hydroxylase (C3H), caffeic acid 3-O-methyltransferase (COMT), cinnamoyl CoA reductase (CCR) and cinnamyl alcohol dehydrogenase (CAD). The three main lignin polymerizations, which form under the catalysis of peroxidase (POD) from monolignol, are: guaiacyl lignin, syringly lignin and hydroxyphenyl lignin (Mottiar et al., 2016).

The transcriptome is an important tool for studying gene expression in organisms, and the metabolome is the basis for and direct manifestation of an organism’s phenotype. A combined analysis of the transcriptome and metabolome can be used to study the internal changes of organisms at different levels and examine the functions and regulatory mechanisms of biomolecules more systematically and comprehensively. A combined analysis of the transcriptome and metabolome has been used to study *Sapium sebiferum* (Yang et al., 2015), *Ginkgo biloba* (Wang et al., 2016), *Pinus tabuliformis* (Niu et al., 2013), *Pinus radiata* (Escandón et al., 2018), and poplar (*Populus* L.) (Chhana et al., 2019). In plants, this method is used to understand various biological traits and phenomena.

Research on the mechanism of lignin synthesis is of great significance for understanding plant growth and environmental adaptability. Many experts and scholars both in China and worldwide have conducted research on this mechanism for many years, such as in *Oryza sativa* (Jung et al., 2013), *Punica granatum* (Zhang et al., 2017) and poplar (Zhong et al., 2000). To date, there have been no reports on the use of multiple omics techniques to understand biological phenomena in *P. orientalis*. This study explored both the distribution of lignin and changes in lignin content in *P. orientalis* by detecting dynamic changes in the roots, stems and leaves of *P. orientalis* and in the stem anatomy. This study also inferred the mechanisms underlying the synthesis and regulation of *P. orientalis* lignin from the perspective of transcription and metabolism. The results of this study are important to regulating the lignin content of *P. orientalis* and improving the adaptability and resistance of this plant.

## Material and Methods

### Plant materials

One-year-old seedlings of a *P. orientalis* half-sib family preserved in the Forestry Experimental Station of Shandong Agricultural University in Tai’an city, Shandong Province, were used as the research objects. The mother tree is located in the North Campus of Shandong Agricultural University. Seeds were collected in November 2018 and sown in March 2019. Single plants with good growth, uniformity and no pests or diseases were selected as the test objects. The annual change of seedling development was observed (Fig. S1).

### Lignin content measurement

*P. orientalis* plants with consistent growth, no disease and insect pests, and good growth conditions were chosen, and roots (CR1-CR9), stems (CS1-CS9) and leaves (CL1-CL9) were sampled on 1st June, 15th June, 1st July, 15th July, 1st August, 15th August, 1st September, 1st October and 1st November, respectively. The samples were collected and taken into the laboratory for lignin content determination. Five individual plants were collected and all measurements were taken three times in each time period.

To extract the lignin, 50 mg of dried sample was ground and homogenized in one mL 80% ethanol, and then placed in a water bath for 10 min at 95 °C. After cooling to room temperature, the sample was centrifuged at 8,000 rpm for 10 min at 25 °C. The precipitate was again subjected to the same extraction procedure with 1.5 mL of 80% ethanol. After that, the saved precipitate was added to one mL extraction reagent (to remove starch) and allowed to stand for 12 h and then centrifuged at 8,000 rpm for 10 min at 25 °C. After removing the supernatant, the precipitate was treated with one mL acetone and centrifuged at 8,000 rpm for 10 min at 25 °C. The saved precipitate, the cell wall material (CWM), was dried in an oven at 85 °C to constant weight and then stored for lignin content analysis. The lignin content was determined by a microplate reader at 280 nm at 25 °C according to a previous study (Shimada, Tsuyama & Kamei, 2019).

### Hard tissue slice

For microscopic observation, a paraffin-based technique was performed in the stem anatomy using the hard tissue slice method as described in a previous study, with slight modifications (Hamann, Smets & Lens, 2011). A total of ten stem samples were taken at every two internodes from the first internode at the top of *P. orientalis* seedling. After fixation, dehydration, dipping, and embedding, the stem tissues were sliced to 10 µm using a microtome and baked overnight at 60 °C. The obtained slices were mordanted with 1% phosphomolybdic acid, and then stained with 1% phloroglucinol stain A for 2 min. The mounted stem samples were stored on a cover glass, and pictures were taken within 3 min.

### Transcriptome analyses

We selected three developmental stages during the annual growth of *P. orientalis* based on the lignin content and hard tissue slice analyses. Samples were collected from the junctions of the rhizomes of individual *P. orientalis* plants above the 2-cm stem segments on 15th July (RM1), 1st September (RM2), and 1st November (RM3). There were three biological replicates for each time point.

The total RNA was extracted from the nine samples, and the concentration and integrity of the total RNA were detected using 1% agarose gels and a Nanodrop 2000 Spectrophotometer (Thermo Fisher Scientific, Waltham, MA, USA). According to the methods previously described by Wu et al. (2020), nine cDNA libraries were constructed and normalized and then sequenced on the Illumina HiSeq platform. The sequence data were deposited in the NCBI Sequence Read Archive (SRA) under accession numbers SRR18002871, SRR18002870, SRR18002869, SRR18002868, SRR18002867, SRR18002866, SRR18002865, SRR18002864 and SRR1800286. The raw data were processed by removing the low-quality sequences (reads with more than 50% Q < 19 bases), the adaptor-pollute sequences, and sequences with ambiguous base reads accounting for more than 5%. After filtration, the clean reads were assembled into unigenes by the Trinity software and then used to analyze functional annotation and the gene expression level. Unigenes with *q*-values ≤0.05 and a fold change ≥1 were identified as differentially expressed genes (DEGs). The GO annotation and KEGG enrichment of DEGs were implemented using the hypergeometric test, in which the *p*-value was calculated and adjusted as a *q*-value: genes with *q*-values of <0.05 were considered to be significantly enriched.

### Metabolome analyses

Metabolome sample collection was performed in the same manner as transcriptome collection, and nine samples were vacuum freeze-dried and ground to powder with a grinder at 35 Hz for 1.5 min. A total of 100 mg of the resulting powder was dissolved in 1.0 mL of extract. The dissolved sample was placed in a refrigerator overnight at 4 °C, during which it was vortexed three times to increase the extraction rate. After centrifugation at 10,000 rpm for 10 min, the supernatant was aspirated, and the sample was filtered with a microporous membrane (0.22 µm pore size). Then, the sample was saved in a sample bottle for metabolomic analysis. The LC-MS/MS conditions and data collection were performed using methods previously described by Wu et al. (2021). Using Analyst 1.6.1 software to process the mass spectrum data, based on the local database, the metabolites of the sample were analyzed qualitatively and quantitatively by mass spectrometry. Based on the results of the multivariate analysis of the variable importance projection (VIP) of the OPLS-DA model, the metabolites that differed in each sample were chosen for screening. The differential metabolites (DMs) were then selected using the values of fold change and VIP according to a previous study (Wu et al., 2021). The KEGG database was used to perform a functional annotation and enrichment analysis of differential metabolites.

### Combined analyses

A correlation analysis was performed on the genes and metabolites detected by the combined comparison of differences. The cor program in R was used to calculate the Pearson correlation coefficients of the genes and metabolites, and nine-quadrant graphs and correlation scatter plots were used to show the genes and metabolites with Pearson correlation coefficients greater than 0.8 and the difference multiples of the genes in each difference group; network graphs were used to show the relationship between the metabolites and genes. The DEGs and DMs in the ko00940 pathway with a correlation greater than 0.8 were selected and used to generate a network diagram of the correlation between the genes and metabolites.

### qRT-PCR validation

Twenty candidate genes related to lignin biosynthesis were selected to verify the expression of DEGs in the transcriptome. Six of these candidate genes were selected to determine the expression level of each candidate gene in different tissues and explore its correlation with lignin biosynthesis. The amplification primers were designed by the Primer Premiere 5.0 software. All primers used in this experiment are listed in Table S1. All reactions and analyses were carried out by SYBR Premix Ex Taq and a Bio-Rad CFX96 thermal cycler according to the manufacturer’s instructions.

### Statistical analysis

Values were expressed as mean  ± SD of three independent experiments. The SAS 9.2 software was used to analyze the variance in lignin content through ANOVA, the R package was used to process and graph the sequencing data, and the Bio-Rad CFX96 software was used to analyze the qRT-PCR data.

## Results

### Changes in lignin content

**Figure 1:**
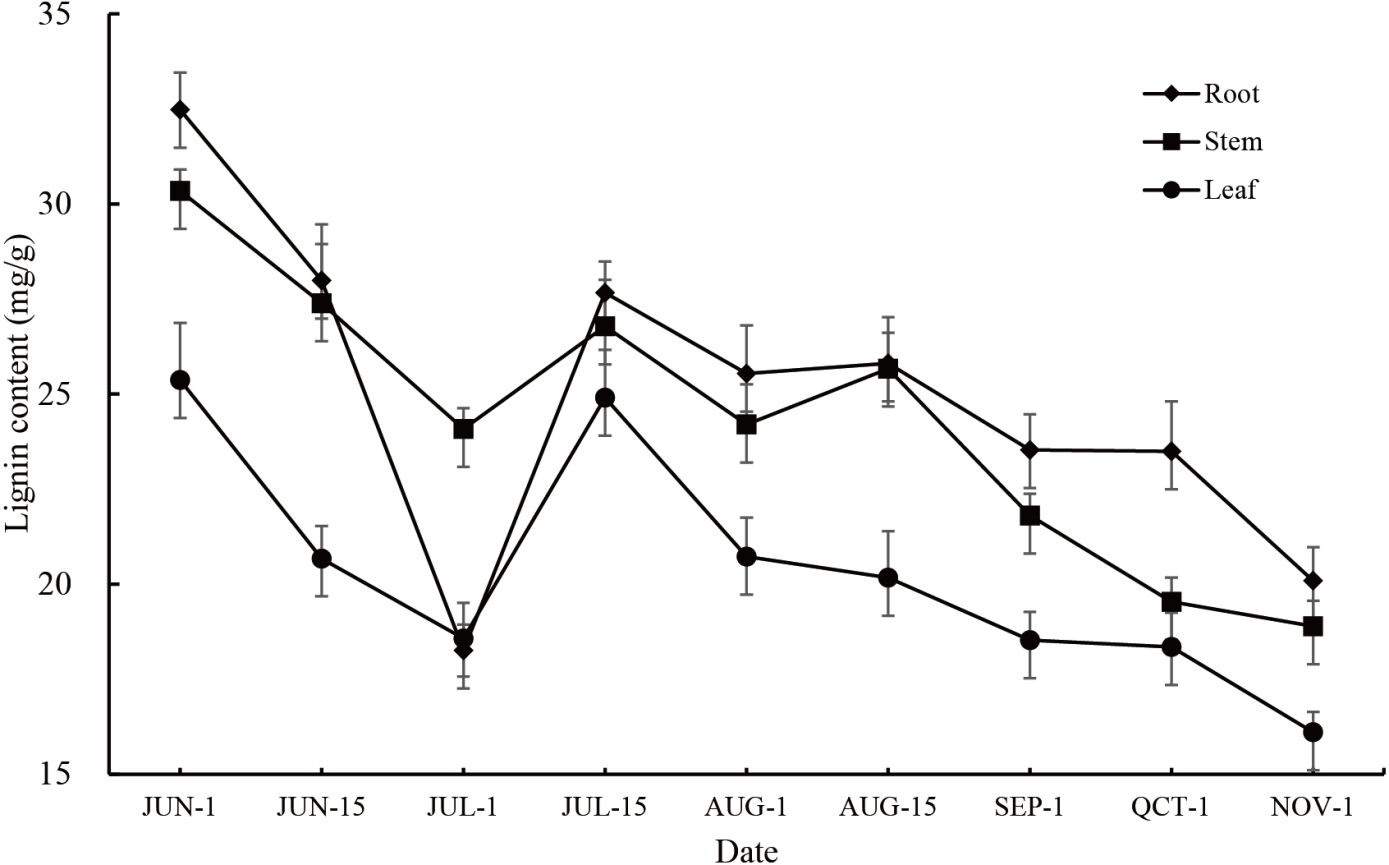
Annual variation in lignin content in different tissues of *P. orientalis*.

The lignin content in the roots, stems and leaves of *P. orientalis* showed large annual changes as well as significant seasonal changes (*P* < 0.01). The change trends in all tissues were consistent, showing that the lignin content gradually decreased from early June to early July, gradually increased from early July to mid-July, and then decreased at middle to later stages of the observation period. During the whole observation period, the root had the highest lignin content except for at the 1st July observation, followed by the stem and leaf (Fig. 1, Table S2).

### Changes in the anatomical characteristics of the stem

**Figure 2:**
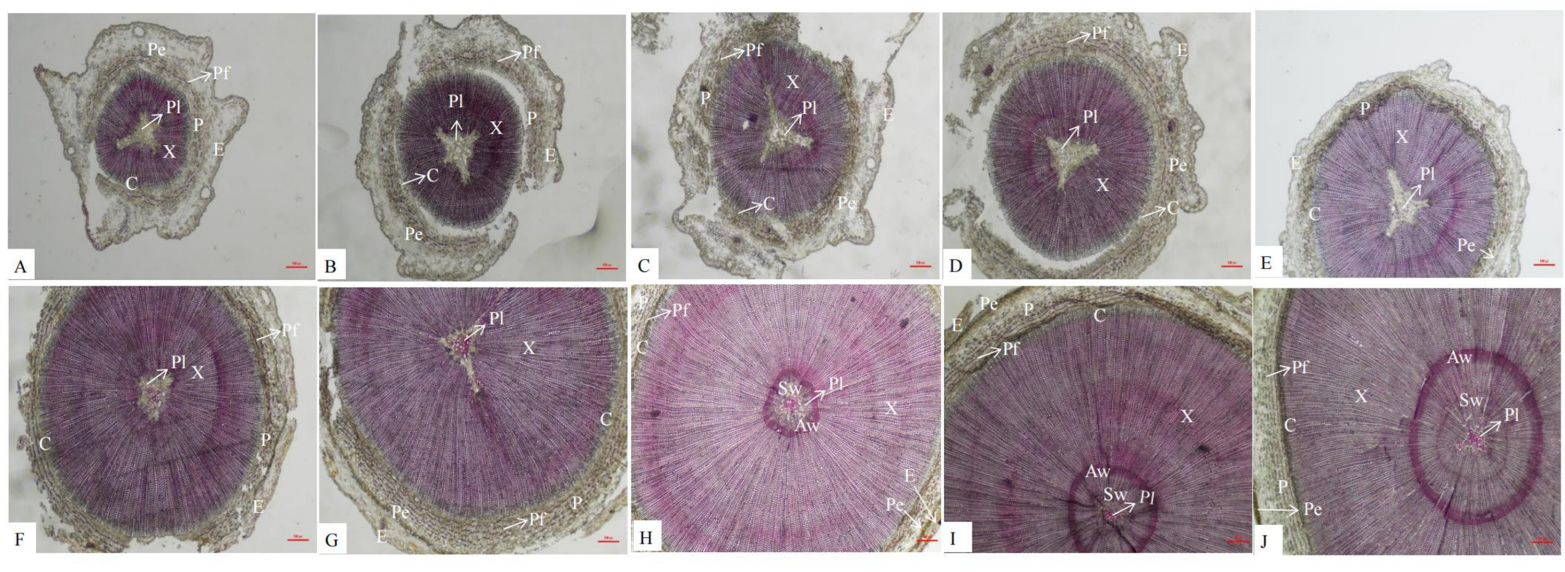
Dynamic change of different internodes of *P. orientalis* stem. (A) The first internode. (B) The second internode. (C) The thirdly internode. (D) The fourth internode. (E) The fifth internode. (F) The sixth internode. (H) The seventh internode. (H); The eighth internode. (I) The ninth internode. (J) The tenth internode. X, xylem, P, phloem, Pe, periderm, C, cambium, Pi, pith, E, epidermis, C, cortex, Pf, phloem fiber.

An anatomical analysis showed that the secondary structures of *P. orientalis* stems, from outside to inside, are the epidermis, pericyte, phloem, cambium, xylem, and pith. Lignin is mainly distributed in the cell walls of the pith, xylem, phloem, perimeter, and epidermis. In the early stage of stem development, there was no lignin at all in the pith, but as the stems continued to develop, the lignin content in the pith gradually increased (Fig. 2).

### Overview of transcriptome and metabolome data

The transcriptome and metabolome sequencing data were filtered and evaluated, and the results showed that the Q30 score of each sample of the transcriptome was above 93% showing that high-quality, clean reads were obtained. There were large differences among the treatments in the metabolite group, but the difference between different replicates of the same treatment was small, and the sample repeatability was high according to a principal component analysis and hierarchical cluster analysis (Fig. S2). High-quality sequencing data provide a guarantee for a subsequent bioinformatics analysis (Tables S3 and S4).

**Figure 3:**
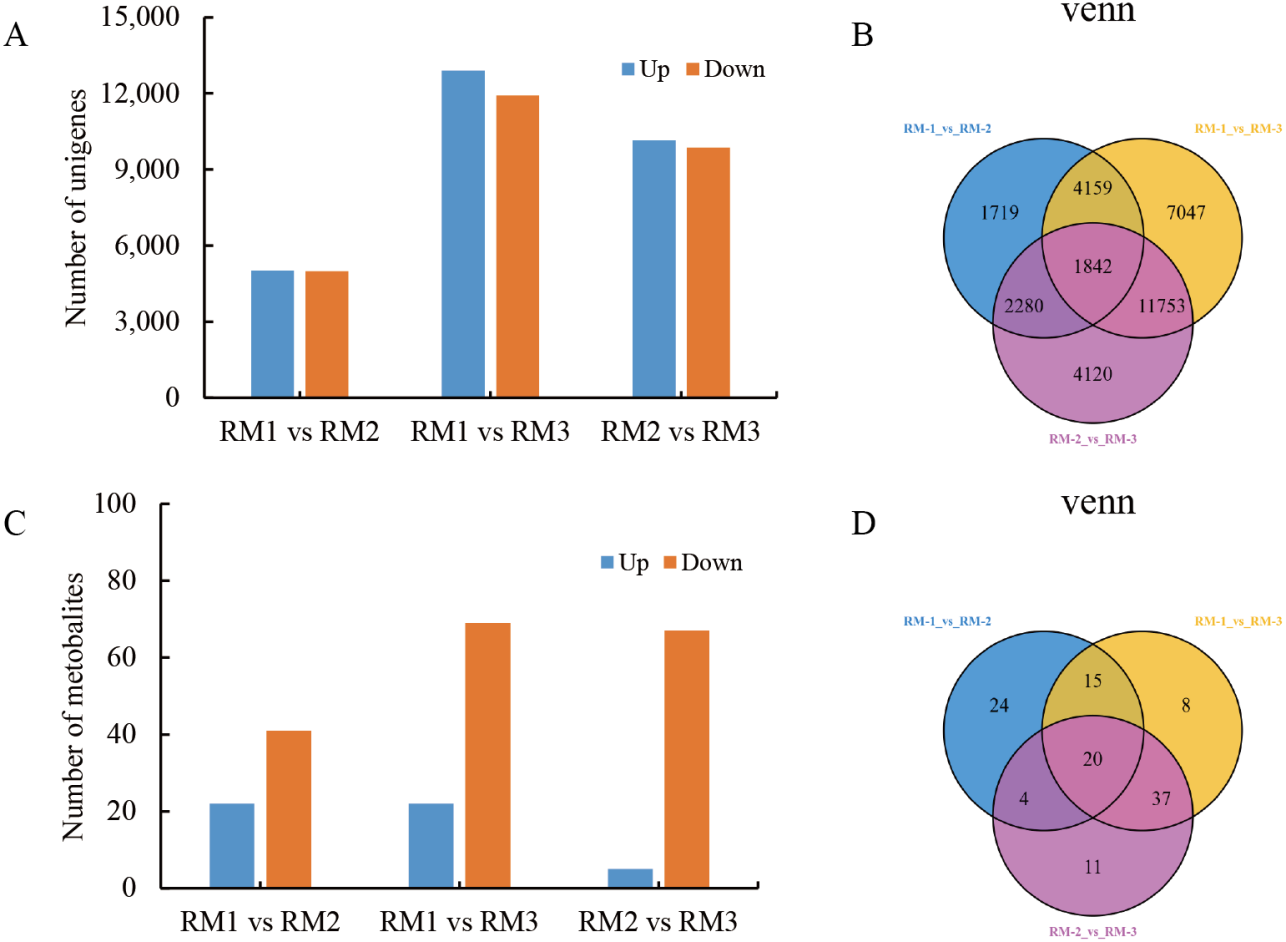
Statistical analysis of DEGs and DMs during *P. orientalis* stem developmen. (A) The number of up-regulated and down-regulated DEGs. (B) The distribution of DEGs at different time points in the transcriptome analysis. (C) The number of up-regulated and down-regulated DMs. (D) The distribution of DMs at different time points in the metabolome analysis.

A large number of DEGs and DMs were detected in each differential comparison combination (Tables S2 and S3). In RM1 *vs* RM2, 10,000 DEGs (5,016 up-regulated and 4,984 down-regulated) and 63 DMs (22 up-regulated and 41 down-regulated) were detected. In RM1 *vs*. RM3, 24,801 DEGs (12,892 up-regulated and 11,909 down-regulated) and 80 DMs (11 up-regulated and 69 down-regulated) were detected. In RM2 *vs*. RM3, 19,995 DEGs (10,143 up-regulated and 9,852 down-regulated) and 72 DMs (5 up-regulated and 67 down-regulated) were detected (Figs. 3A and 3C). A total of 20 DMs and 1,842 DEGs were found to be common among the three comparison combinations (Figs. 3B and 3D).

Twenty DEGs were randomly selected for expression verification by qRT-PCR. The expression levels of the 20 DEGs obtained by qRT-PCR were compared with the FPKM values of the transcriptome sequencing data. Seventeen genes were up-regulated in the high-lignin group: *cluster-44281.111976*, *cluster-44281.66535*, *cluster-44281.83773*, *cluster-44281.47921*, *cluster-44281.38219*, *cluster-44281.91549*, *cluster-44281.69804*, *cluster-68156.0*, *cluster-44281.95346*, *cluster-77994.4*, *cluster-44281.103971*, *cluster-44281.38217*, *cluster-68496.0*, *cluster-44281.111418*, *cluster-44281.131789*, *cluster-44281.131493*, *cluster-44281.111830*, and three genes, *cluster-59398.0*, *cluster-44281.68469* and *cluster-44281.67555*, were up-regulated in the low-lignin group. The FPKM value of each gene in the transcriptome sequencing was consistent with the expression trend obtained by qRT-PCR (Fig. S3, Table S5).

### Screening and functional annotation of DEGs and DMs

For the GO annotation of RM1 *vs.* RM2, the identified DEGs were assigned into 55 sub-categories, included in the three main GO functional categories (Fig. S4), including biological process (BP), cellular component (CC), and molecular function (MF). For the BP category, the three most abundant sub-categories were ‘cellular process’(4,488 DEGs), ‘metabolic process’ (3,879 DEGs), and ‘response to stimulus’ (2,429 DEGs). The majority of DEGs of CC category were assigned into ‘cell’ (5,321 DEGs), ‘cell part’ (5,311 DEGs), and ‘organelle’ (3,689 DEGs). The three most abundant sub-categories which the MF category was divided were ‘Binding’ (4,569 DEGs), ‘catalytic activity’ (3,902 DEGs), and ‘transporter activity’ (604 DEGs). In the groups RM1 *vs.* RM3 and RM2 *vs.* RM3, the identified DEGs were assigned into 56 and 55 sub-categories, respectively, with the similar results for the most abundant pathways enrichment in RM1 *vs.* RM2 (Table S6).

The detected DEGs and DMs were enriched and functionally annotated in the KEGG database (Fig. S5). The results showed that in the KEGG database, the DMs in the RM1 *vs*. RM2 combination were distributed in 27 pathways, the DMs in the RM1 *vs*. RM3 combination were distributed in 14 pathways, and the DMs in the RM2 *vs*. RM3 combination were also distributed in 14 pathways. In these three comparison combinations, the pathway that contained the most differential metabolites was the biosynthesis of secondary metabolites (ko01110), and the pathway with the most DEGs annotations was the metabolic pathway (ko01100) (Table S7).

**Figure 4:**
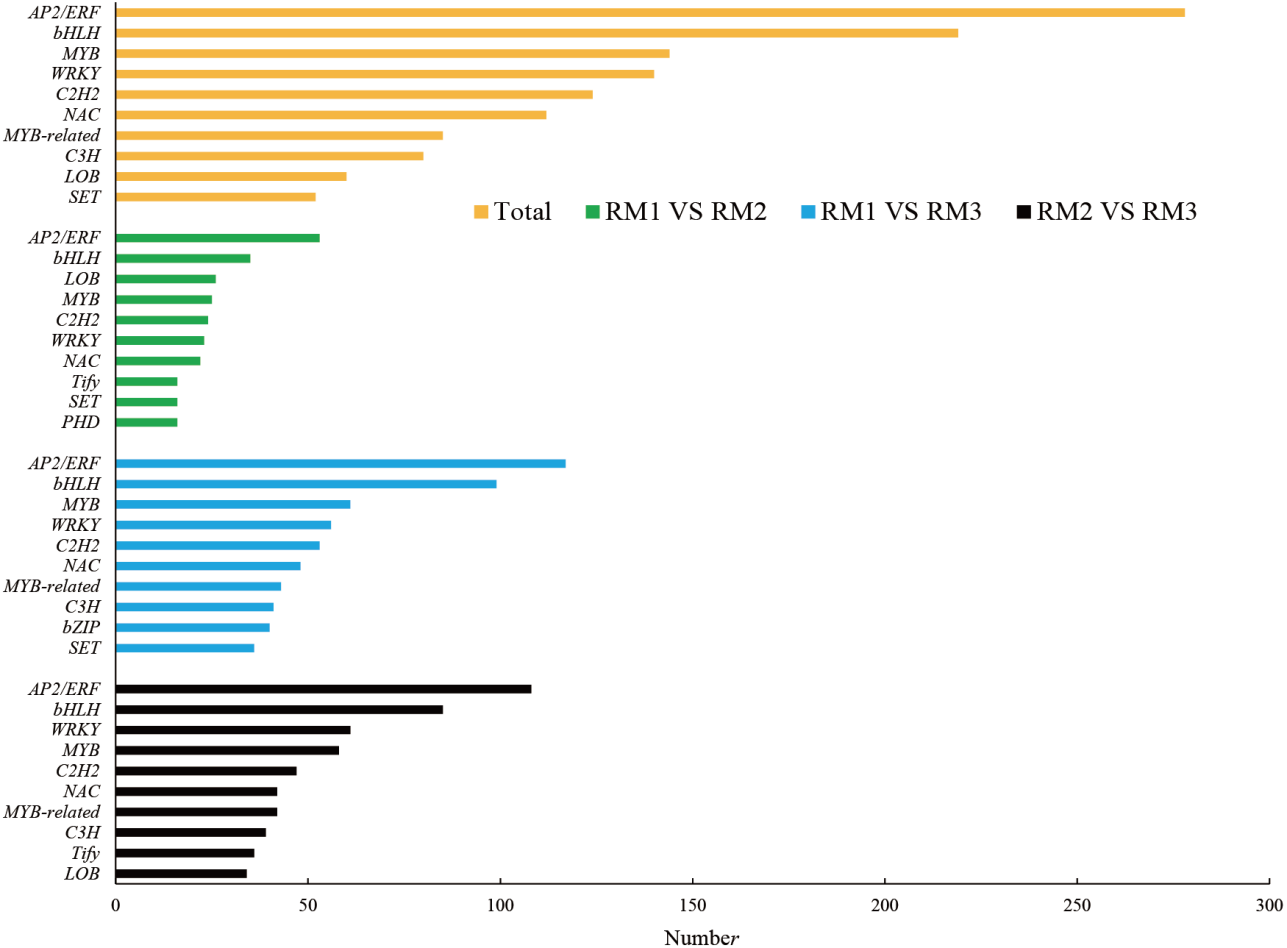
Number of top 10 TF families in each combination.

In ko00940, the main pathway of lignin synthesis, DEGs and DMs with a correlation greater than 0.8 were selected, and seven key enzymes and 24 key DEGs were screened, including 21 positively regulated genes and three negatively regulated genes. Four positively regulated unigenes were identified for 4CL: *cluster-44281.111830*, *cluster-44281.38217*, *cluster-44281.38219*, and *cluster-44281.66535*. The unigene of *cluster-68156.0* showed a positive correlation with COMT and the unigene of *cluster-44281.73529* showed a negative correlation with Caffeoyl-CoA-O-methyltransferase (CCoAOMT). There were five unigenes found to be related to CCR, including four positive unigenes (*cluster-44281.111976*, *cluster-44281.42325*, *cluster-44281.69804* and *cluster-44281.89328*) and one negative unigene (*cluster-44281.67555*). Three positive unigenes (*cluster-44281.103971*, *cluster-44281.95346*, *cluster-59398.0*) and one negative unigene (*cluster-44281.68469*) showed significant correlation with CAD. Seven unigenes (*cluster-68496.0*, *cluster-44281.131493*, *cluster-44281.131789*, *cluster-44281.47921*, *cluster-44281.91549*, *cluster-77994.4*, *cluster-68496.0*) showed a positive correlation with POD while three (*cluster-44281.111418*, *cluster-44281.83773*) showed a positive correlation with phenylalanine ammonialyase (PAL). A total of 2,877 transcription factors (TFs) were enriched in each comparison combination. The largest family was *AP2/ERF* with 278 TFs, accounting for 9.66% of the total, followed by the *bHLH* family (219, 7.61%), the *MYB* family (144, 5.01%), the *WRKY* family (140, 4.87%) and the *C2H2* family (124, 4.31%; Fig. 4).

### Correlation analysis of the genes and metabolites related to lignin metabolism

**Figure 5:**
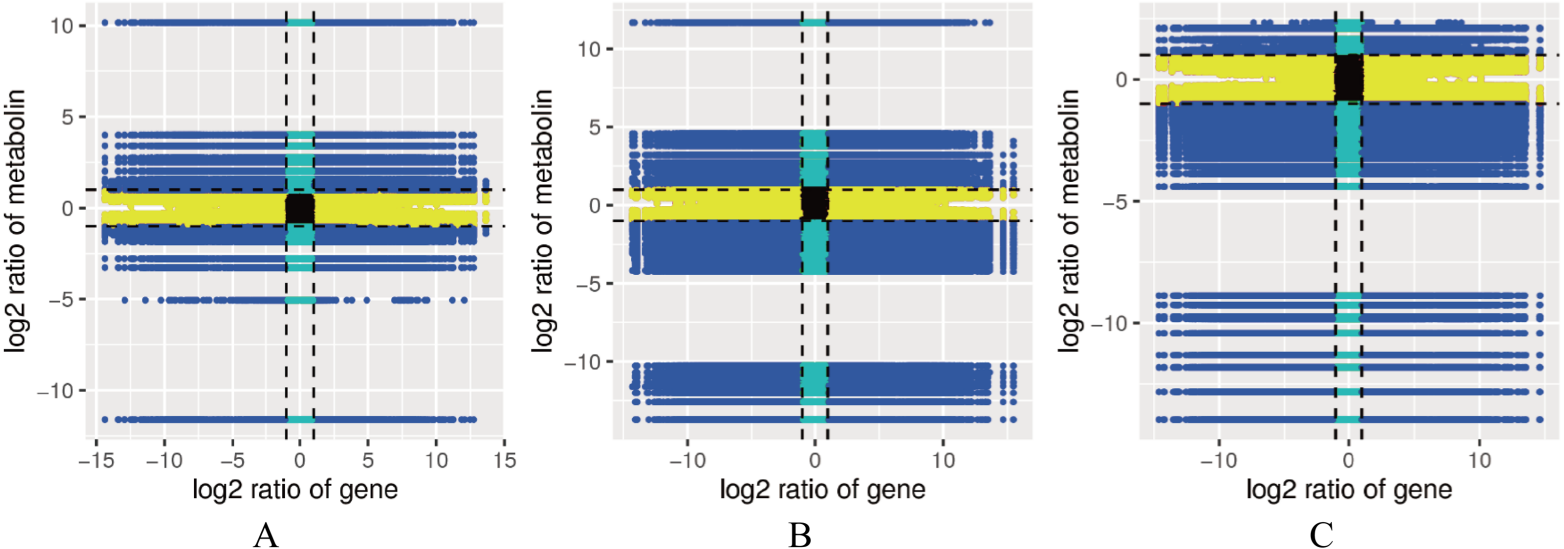
Correlation map between the levels of each gene and metabolite in the control combinations. (A) RM1 *vs* RM2. (B) RM1 *vs* RM3. (C) RM2 *vs* RM3. Quadrant 1, 2 and 4, Genes are negatively correlated with metabolites, and the expression abundance of metabolites is higher than that of genes. Quadrants 3 and 7, The differential expression patterns of genes and metabolites are consistent. Quadrant 5, Genes and metabolites are not differentially expressed. Quadrants 6, 8 and 9, Genes are negatively correlated with metabolites, and the expression abundance of metabolites is lower than that of genes.

A correlation analysis was performed on the genes and metabolites detected in each group, and the cor program in R was used to calculate the Pearson correlation coefficients of the genes and metabolites. The nine-quadrant graph showed the multiples of differences in the gene metabolites with Pearson correlation coefficients greater than 0.8 in each difference group. Black dashed lines were used from left to right and top to bottom to divide the plot into quadrants 1-9 to obtain a large number of consistent gene and metabolite expression patterns (Fig. 5). Multiple enzymes were related to the lignin metabolism process. The genes identified for POD (*cluster-77994.4*, *cluster-44281.91549*, *cluster-44281.56610*, etc.), CCR (*cluster-44281.80743*, *cluster-44281.71185*, *cluster-44281.67555*, etc.), CAD (*cluster-59398.0*, *cluster-44281.95346*, *cluster-44281.68469*, etc.), 4CL (*cluster-44281.66535*, *cluster-44281.38219*, *cluster-44281.38217*, etc.), COMT (*cluster-68156.0*), and CCoAOMT (*cluster-44281.73529*) showed positive correlations with 1-O-galloyl- *β*-D-glucose, 3-indoleacrylic acid, 4-aminobenzoic acid, 6′-O-D-glucosylloganic acid, luteolin-O-rutinoside-O-rhamnoside, olival-4′-O-*β*-D-glucoside and other metabolites (Table S8).

**Figure 6:**
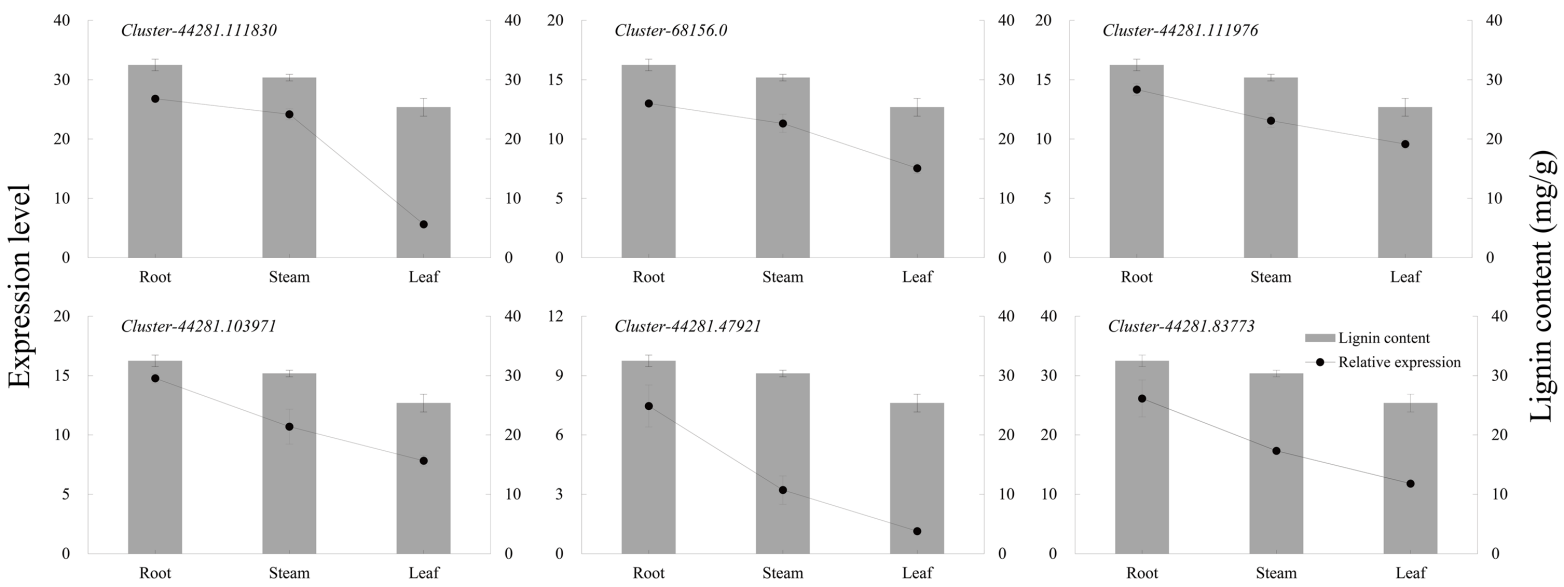
The expression levels of six key genes in lignin biosynthesis in different *P. orientalis* tissues.

The six key genes in the lignin biosynthesis pathway were selected for qRT-PCR expression verification in *P. orientalis* roots, stems, and leaves. The results showed that the expression levels of *cluster-44281.111830* (*4CL*), *cluster-68156.0* (*COMT*), *cluster-44281.111976* (*CCR*), *cluster-44281.103971* (*CAD*), *cluster-44281.47921* (*POD*), and *cluster-44281.83773* (*PAL*) were highest in the roots, followed by the stems, with the lowest expression observed in the leaves. Gene expression levels were positively correlated with lignin content (Fig. 6, Table S9).

### Regulatory network of key factors related to the lignin biosynthesis pathway

**Figure 7:**
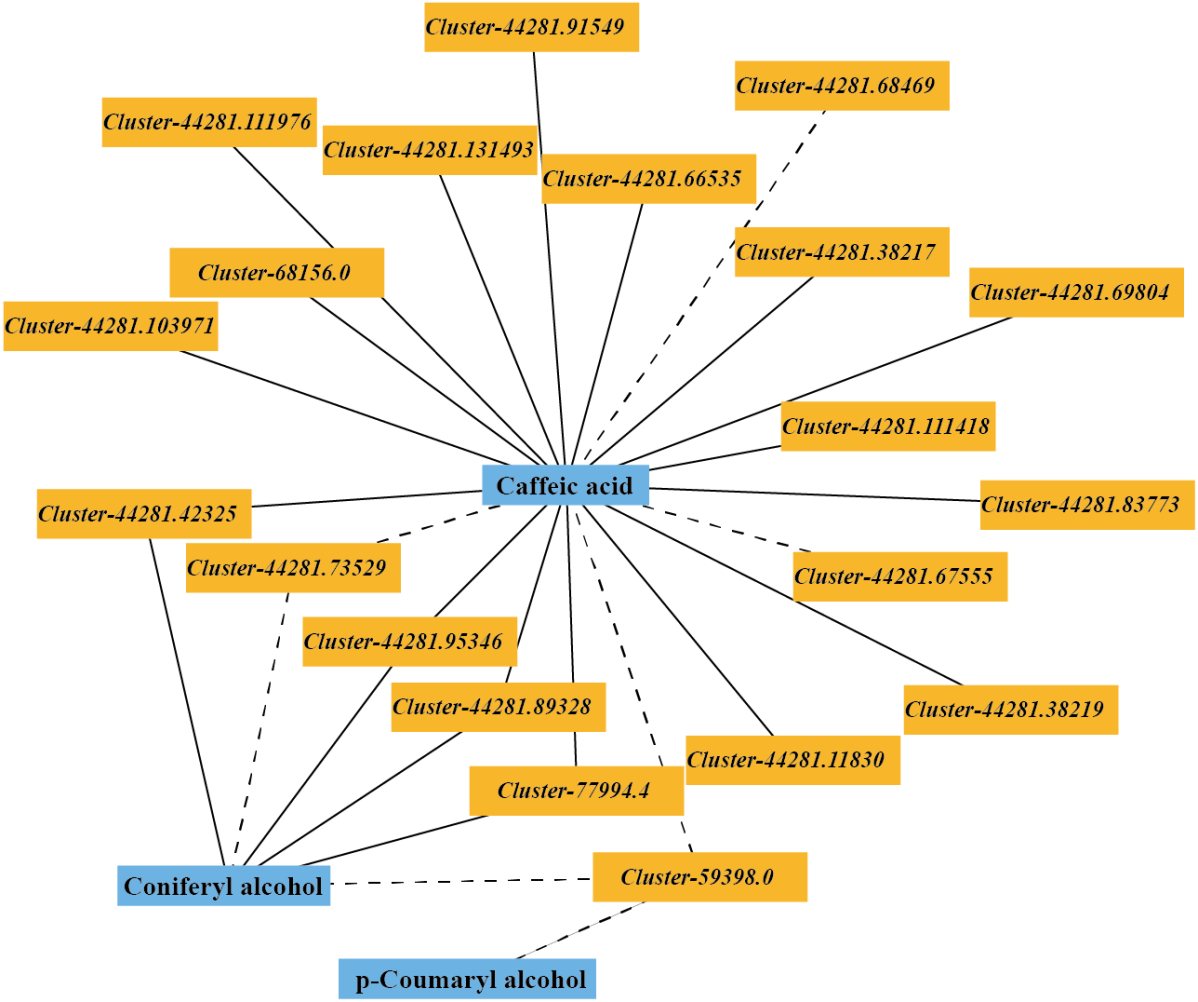
Correlation network of major metabolites and genes in the lignin metabolism pathway.

The DEGs and DMs in the ko00940 pathway with a correlation greater than 0.8 were selected to draw a correlation network diagram of the differentially expressed genes and differential metabolites. In the ko00940 pathway, coniferyl alcohol, p-coumaryl alcohol, caffeic acid, *cluster-44281.42325* (*CCR*), *cluster-44281.89328* (*CCR*), *cluster-44281.73529* (*CCoAOMT*), *cluster-44281.95346* (*CAD*), *cluster-59398.0* (*CAD*), *cluster-77994.4* (*POD*), *cluster-44281.83773* (*PAL*), *cluster-44281.66535* (*4CL*), *cluster-68156.0* (*COMT*) and other genes have complex regulatory relationships (Fig. 7).

**Figure 8:**
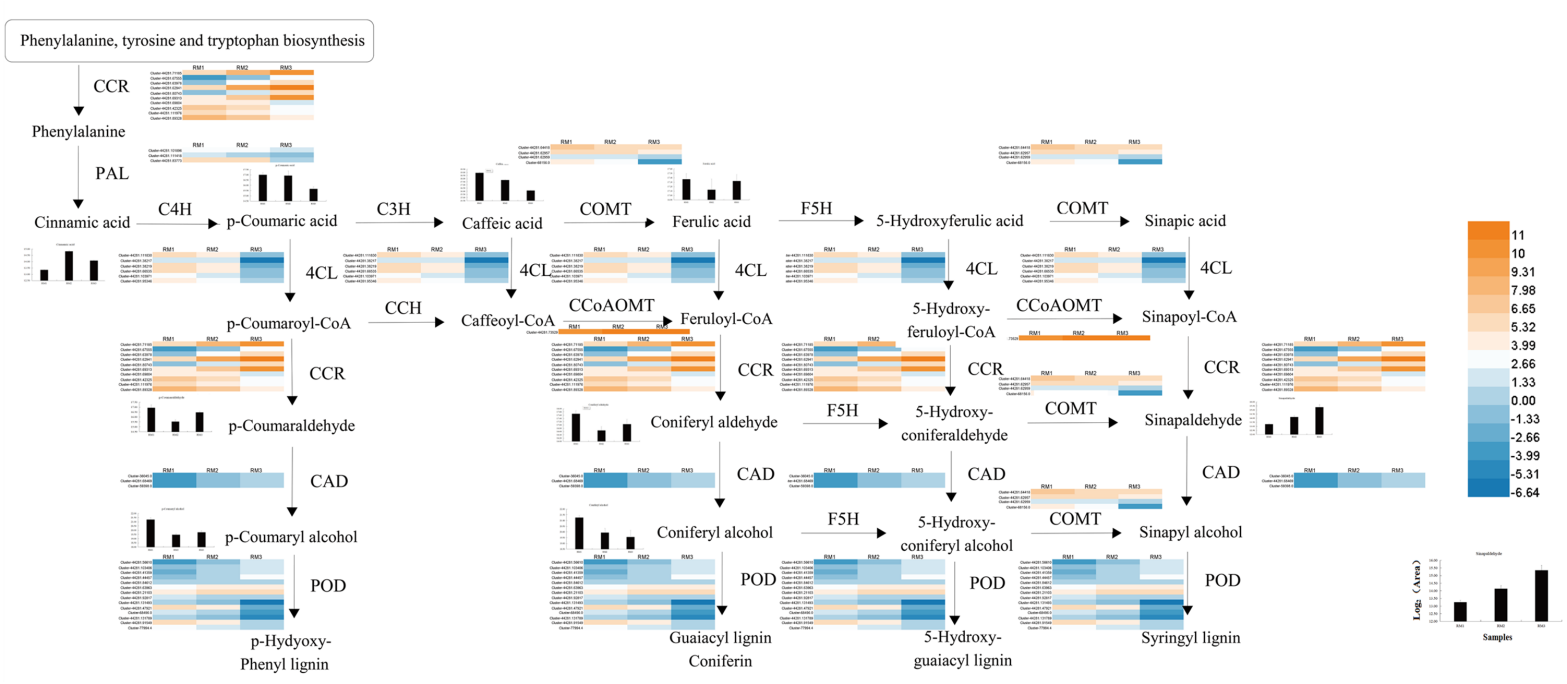
Key regulatory pathways of lignin metabolism in *P. orientalis*. PAL, Phenylalanine ammonia-lyase; C3H, Coumarate 3-hydroxylase; C4H, Cinnamate 4-hydroxylase; COMT, Caffeic acid/5-hydroxy coniferaldehyde O-methyltransferase; 4CL, 4-hydroxycinamate CoA ligase; F5H, Ferulate-5-hydroxylase; CCoAOMT, Caffeoyl-CoA O-methyltransferase; CAD, Cinnamyl alcohol dehydrogenase; CCR, Cinnamoyl-CoA reductase.

A diagram of the key regulatory pathways for lignin biosynthesis in *P. orientalis* stems was drawn based on the results of transcriptome and metabolome sequencing and a bioinformatics analysis, as shown in Fig. 8. In the figure, the differential expression heat map of the key genes and the histogram of the differential expression of metabolites are marked. The figure shows how phenylalanine finally generates three types of lignin, namely, G-lignin, S-lignin and H-lignin, under the catalysis of multiple genes and enzymes.

## Discussion

### Spatiotemporal and tissue specificity of lignin content in *P. orientalis*

Lignin content changes in the roots, stems, and leaves of *P. orientalis* were consistent among the different periods. These changes could be roughly divided into three stages with each stage presenting different characteristics. It is speculated that the reason for the changes in lignin content is that lignin is mainly present in the cell wall (Acemi, 2020), and with cell division and growth, the relative content of lignin changes significantly. In the first (early June to early July) and third stages (mid-July until November), *P. ori entalis* cells mainly undergo elongation. The specific surface area of the cell wall gradually decreases as the cell wall gradually increases, making the lignin content of *P. orientalis* decrease. In the second stage (early July to mid-July), *P. orientalis* cells mainly proliferate and grow. The cells continue to divide into new cells. These newly divided cells are small in size and have a large cell wall surface area, so the lignin content in this stage increases continuously. This study also found that the lignin content in the roots, stems and leaves of *P. orientalis* during the same periods also had extremely significant differences. The lignin content from early June to mid-June and mid-July to early November was highest in the root, followed by the stem with the leaf having the lowest lignin content. This is mainly because the volume of lignified tissue in the root is higher than that in the stem and leaf (Sharma et al., 2020). In addition, the root system growth of the seedling comes earlier than stem and leaf growth. This study found that the relative content of lignin in the roots decreased more sharply in the early growth of one-year-old seedlings of *P. orientalis*, which may be related to the characteristics of the seedling growth process. The expansion of the roots, caused by the elongation and growth of cells, leads to an increase in cell surface area, and a decrease in lignin content in the roots. In addition, the growth of other tissues also depends on the secondary metabolites absorbed and accumulated by the roots, leading to a larger decline of lignin in the roots than that seen in other tissues.

There are also differences in the distribution of lignin among different species and types of lignin. In the stems of *P. orientalis*, lignin is mainly distributed in the cell walls of the xylem, phloem, epidermis, pericytes and pith. In sorghum stems (Deng, Guan & Liu, 2014), lignin is mainly distributed in the cell walls of the epidermis, mechanical tissue, xylem and vascular bundle sheath, similar to the distribution of lignin in *P. orientalis* stems. However, in a study by Meng et al. (2016), it was found that there was no lignin deposition in the phloem of the jujube fruit wrapping the vessel. Similarly, there is no lignin in the pith of the stems of *P. orientalis*. As the lignification degree of the stems of *P. orientalis* increases, lignin begins to appear in the center of the pith, and there is no lignin in the pith cells near the xylem. However, Zhang et al. (2018) found that the parenchyma cells of the pith near the xylem of alfalfa began to lignify and then expand to the center of the pith.

### Transcription and metabolic mechanism of lignin biosynthesis in the stem of *P. orientalis*

The synthesis of lignin in plants originates from the metabolism of phenylpropane, which is initiated by the formation of cinnamic acid from phenylalanine under the catalytic action of PAL. Three different lignin species are generated through a series of hydroxylation, methylation and reduction reactions in of C4H, 4CL, CCR, CAD, COMT, CCoAOMT, POD and other enzymes (Hu et al., 2017).

The 4CL enzyme plays an important role in the biosynthesis of lignin (Lavhale, Kalunke & Giri, 2018). In this study, multiple genes regulating the activity of 4CL were found, and they were all up-regulated in the high-lignin group. A previous study (Chen et al., 2019) found that the lignin content in rapeseed with the expression of the exogenous 4CL gene was significantly increased, which is consistent with the conclusion of this study. Phenylalanine ammonia-lyase is the first rate-limiting enzyme in the lignin synthesis pathway, and the level of PAL expression directly affects the lignin synthesis pathway. Studies have shown that inhibiting the activity of PAL leads to a significant decrease in the lignin content of plants, which leads to abnormal plant growth. The results of Wu et al. (2008) showed that the lignin content of bamboo shoots increased during storage, which was accompanied by an increase in PAL and POD activities. In this study, a number of up-regulated genes related to PAL and POD (*cluster-44281.83773*, *cluster-44281.111418*, *cluster-77994.4*, *cluster-44281.47921*, etc.) were enriched in the high-lignin group, which our findings verified. CCR and CAD are key enzymes that regulate the downstream lignin-specific synthesis pathway: CCR controls the key step of carbon entering the lignin metabolic synthesis pathway (Xue et al., 2015) and CAD catalyzes the last step of lignin synthesis. The qRT-PCR verification results in this study showed that the expression of CCR and CAD was positively correlated with lignin content in *P. orientalis*. This is consistent with the research conclusions of Cheng et al. (2017), Tamagnone et al. (1998), and Baghdady et al. (2006). Research also shows that COMT usually has a synergistic effect with CCoAOMT (Hu et al., 2017). Zhao & Dixon (2011) constructed monovalent and bivalent antisense expression vectors for COMT and CCoAOMT*.* The results showed that when the two genes were inhibited at the same time, the lignin content decreased further. Separate from angiosperms such as *Populus*, the metabolic process of lignin in *P. orientalis* creates typical gymnosperms. Through metabonomics detection, we found that phenylalanine, catalyzed by enzymes such as 4CL, CCR and CAD, does not form H-lignin and S-lignin in the stem of *P. orientalis*, but forms G-lignin, which is also the most common lignin type in gymnosperms. During the study, we also detected a small amount of sinapaldehyde, which is a precursor of S-lignin synthesis. S-lignin sythesis may be related to the significantly up-regulated expression of CCoAOMT and COMT, which promote the transformation of Caffeoyl-CoA to sinapaldehyde.

Lignin metabolism is regulated by multiple TFs. Some studies have shown that the TFs of *MYB* (Geng et al., 2020; Zhong, Richardson & Ye, 2007; Jiao et al., 2019; Ko et al., 2014), *NAC* (Rogers & Campbell, 2004) and other families play important roles in the secondary wall synthesis of *Populus* and *Arabidopsis*. In this study, a large number of *bHLH*, *MYB*, *NAC* and other family genes were enriched in each comparative combination. It is worth noting that among the different comparative combinations, the most abundant transcription factor family was *AP2/ERF*, which plays an important regulatory role in plant growth and development and in response to biotic and abiotic stress. The large enrichment of TFs in the process of lignin metabolism may be closely related to the strong adaptability of *P. orientalis* to arid and barren mountain environments.

## Conclusions

In this study, lignin content showed “down-up-down” trends in different tissues of *P. orientalis*. In the stem, lignin mainly existed in the xylem. A large number of DEGs and DMs were detected in each differential comparison combination, with 10,000 DEGs and 63 DMs in RM1 *vs*. RM2, 24,801 DEGs and 80 DMs in RM1 *vs*. RM3, and 19,995 DEGs and 72 DMs in RM2 *vs*. RM3, respectively. A total of 1,842 DEGs and 20 DMs were found to be common among the three comparison combinations. A total of 2,877 TFs were enriched, mainly in the *AP2/ERF* family. Using a combined analysis, seven key enzymes and the 20 key genes involved in the lignin synthesis of *P. orientalis* lignin were selected, and then the regulatory mechanism of lignin synthesis was constructed. This is the first comprehensive analysis of the transcriptome and metabolome for genes and TFs related to lignin synthesis in *P. orientalis* stems. The results of this study will provide a foundation for subsequent gene function verification and the mining of stress resistance genes important to *P. orientalis* forestation in arid and barren mountainous areas.

##  Supplemental Information

10.7717/peerj.14172/supp-1 Figure S1 Changes of morphology during *P. orientalis* seedling development

10.7717/peerj.14172/supp-2 Figure S2 Quality control of samples from biological material (A) Principal component analysis (PCA) of transcriptome analysis. (B) Hierarchical cluster analysis (HCA) of transcriptome analysis. (C) PCA of metabolome analysis. (D) HCA of metabolome analysis.

10.7717/peerj.14172/supp-3 Figure S3 Verification of 20 key differentially expressed genes

10.7717/peerj.14172/supp-4 Figure S4 GO annotation of different expressed unigenes (DEGs) in each comparative combination (A) GO annotation of DEGs of RM1 vs RM2. (B) GO annotation of DEGs of RM1 vs RM3. (C) GO annotation of DEGs of RM2 vs RM3.

10.7717/peerj.14172/supp-5 Figure S5 KEGG enrichment map of different factors in each comparative combination (A) KEGG enrichment of DEGs of RM1 vs RM2. (B) KEGG enrichment of DEGs of RM1 vs RM3. (C) KEGG enrichment of DEGs of RM2 vs RM3. (D) KEGG enrichment of different metabolites (DMs) of RM1 vs RM2. (E) KEGG enrichment of DMs of RM1 vs RM3. (F) KEGG enrichment of DMs of RM2 vs RM3.

10.7717/peerj.14172/supp-6 Table S1 The qRT-PCR primer sequences

10.7717/peerj.14172/supp-7 Table S2 The lignin content in different tissues of *P. orientalis*

10.7717/peerj.14172/supp-8 Table S3 The list of obtained unigenes data

10.7717/peerj.14172/supp-9 Table S4 The list of secondary metabolomics data

10.7717/peerj.14172/supp-10 Table S5 The gene expression in the stem measured by qRT-PCR

10.7717/peerj.14172/supp-11 Table S6 The list of GO annotation of DEGs

10.7717/peerj.14172/supp-12 Table S7 The list of KEGG analysis of DMs and DEGs

10.7717/peerj.14172/supp-13 Table S8 Correlation table of lignin biosynthesis-related differentially expressed genes and metabolites

10.7717/peerj.14172/supp-14 Table S9 The gene expression in different tissues measured by qRT-PCR

## Additional Information and Declarations

### DNA Deposition

The following information was supplied regarding the deposition of DNA sequences: The Sequence data are available in the NCBI Sequence Read Archive (SRA): SRR18002871, SRR18002870, SRR18002869, SRR18002868, SRR18002867, SRR18002866, SRR18002865, SRR18002864, SRR1800286.

### Data Availability

The following information was supplied regarding data availability: The raw measurements are available in the Supplemental Files.
