# Supplementary figures and images for: Transcriptome and metabolome analyses of lignin biosynthesis mechanism of Platycladus orientalis
Source: PeerJ. 2022 Nov 2;10:e14172. doi: 10.7717/peerj.14172 (PMC9636869; doi:10.7717/peerj.14172)

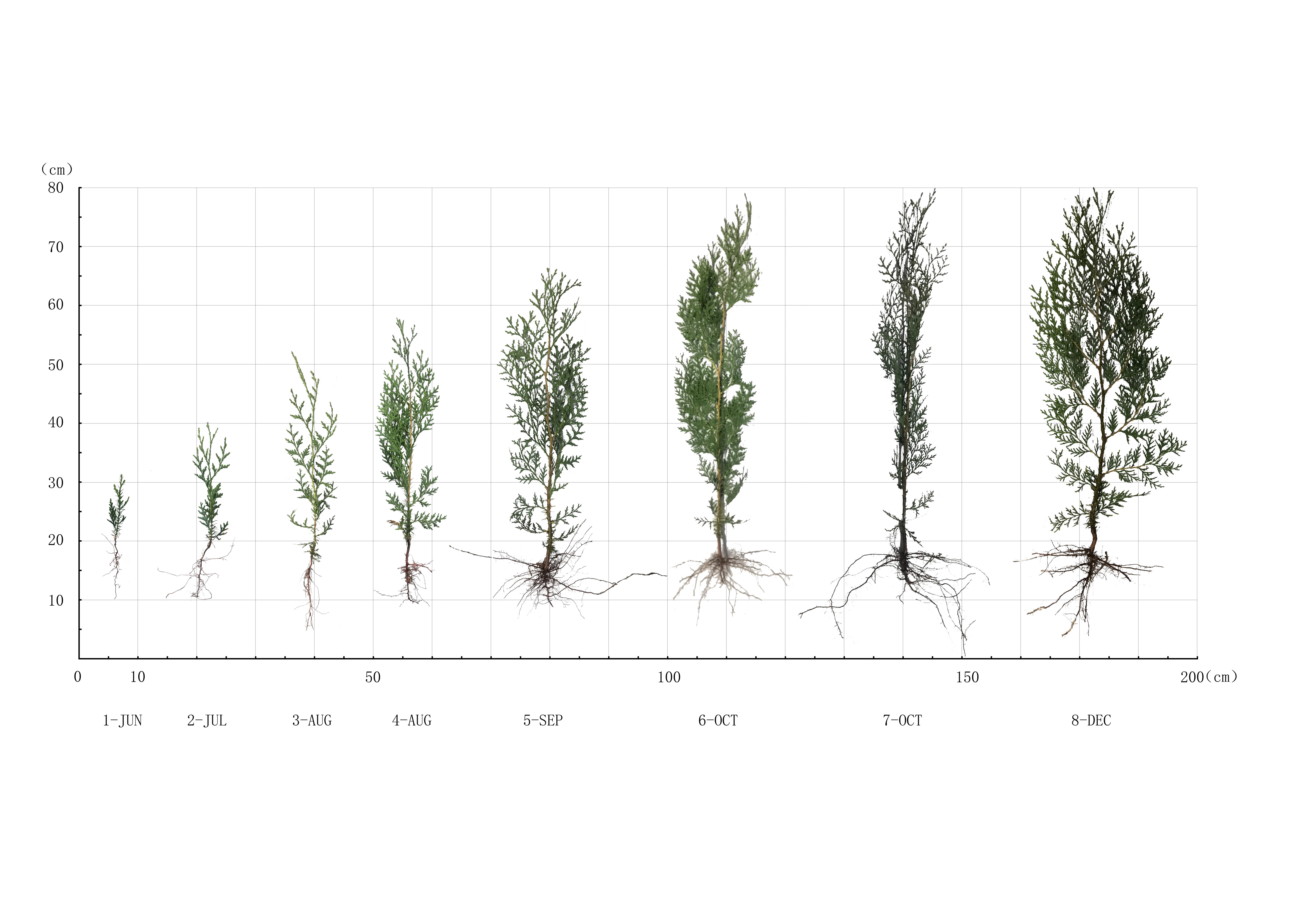

Supplement: Figure S1 [file peerj-10-14172-s001.jpg]

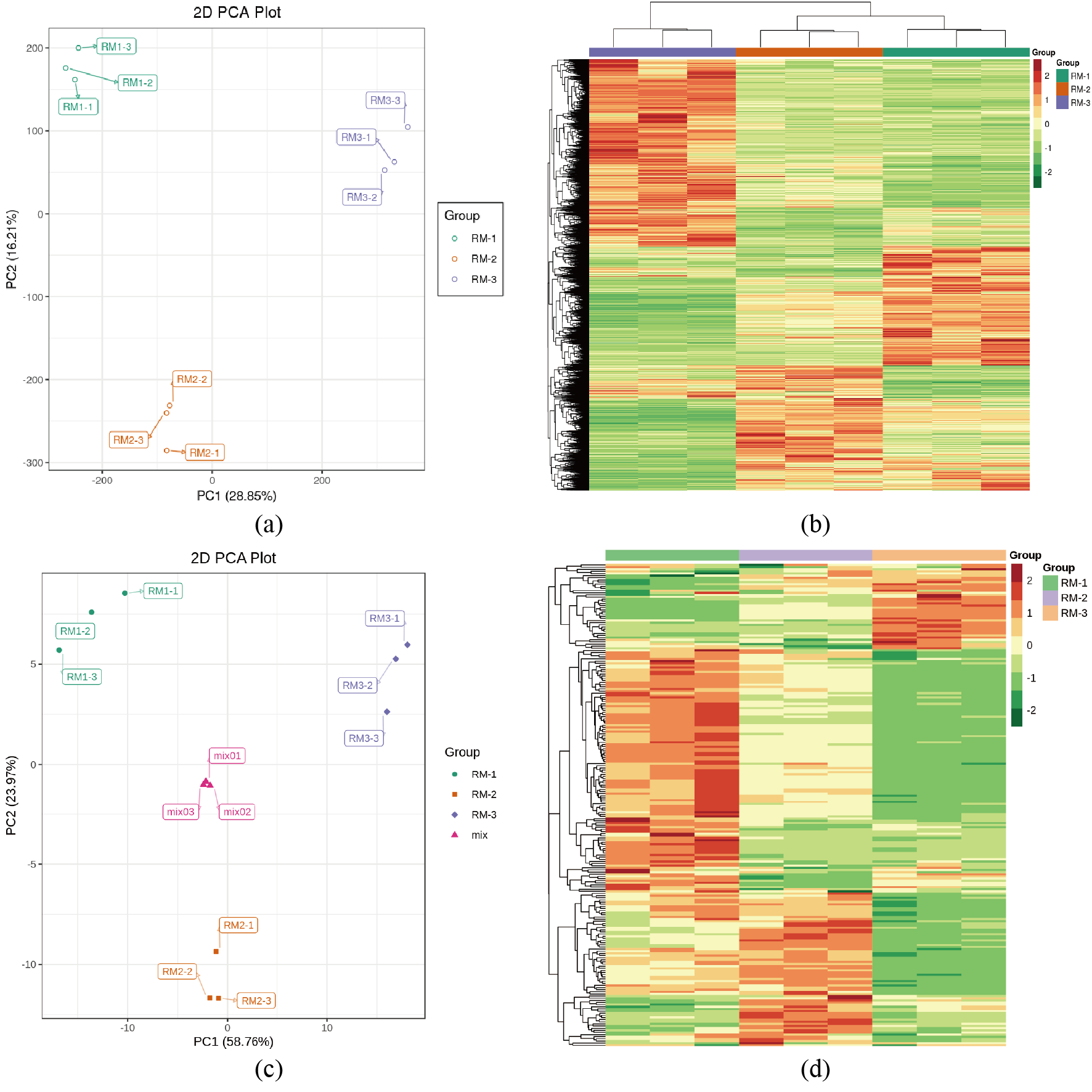

Supplement: Figure S2 — (A) Principal component analysis (PCA) of transcriptome analysis. (B) Hierarchical cluster analysis (HCA) of transcriptome analysis. (C) PCA of metabolome analysis. (D) HCA of metabolome analysis. [file peerj-10-14172-s002.png]

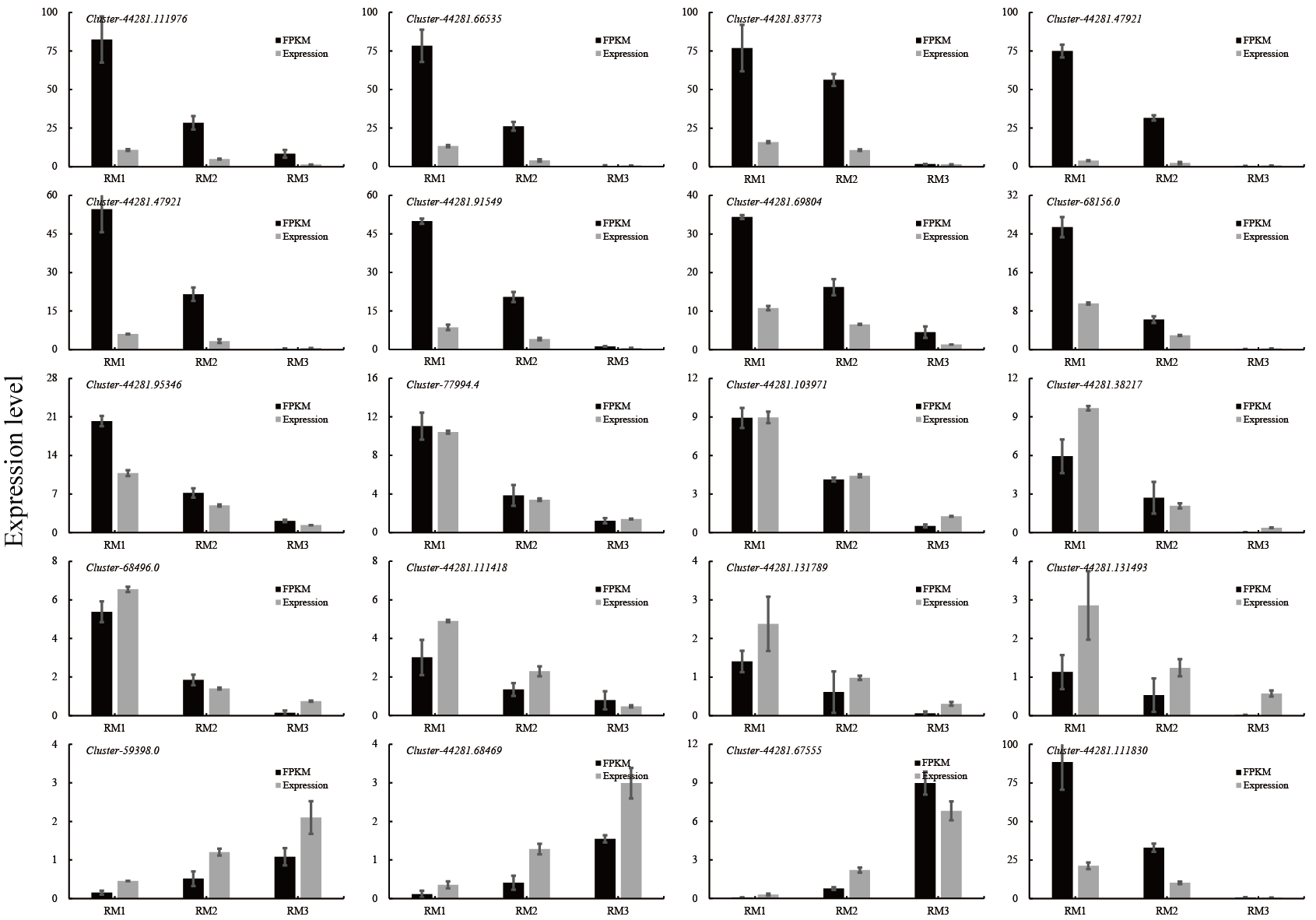

Supplement: Figure S3 [file peerj-10-14172-s003.png]

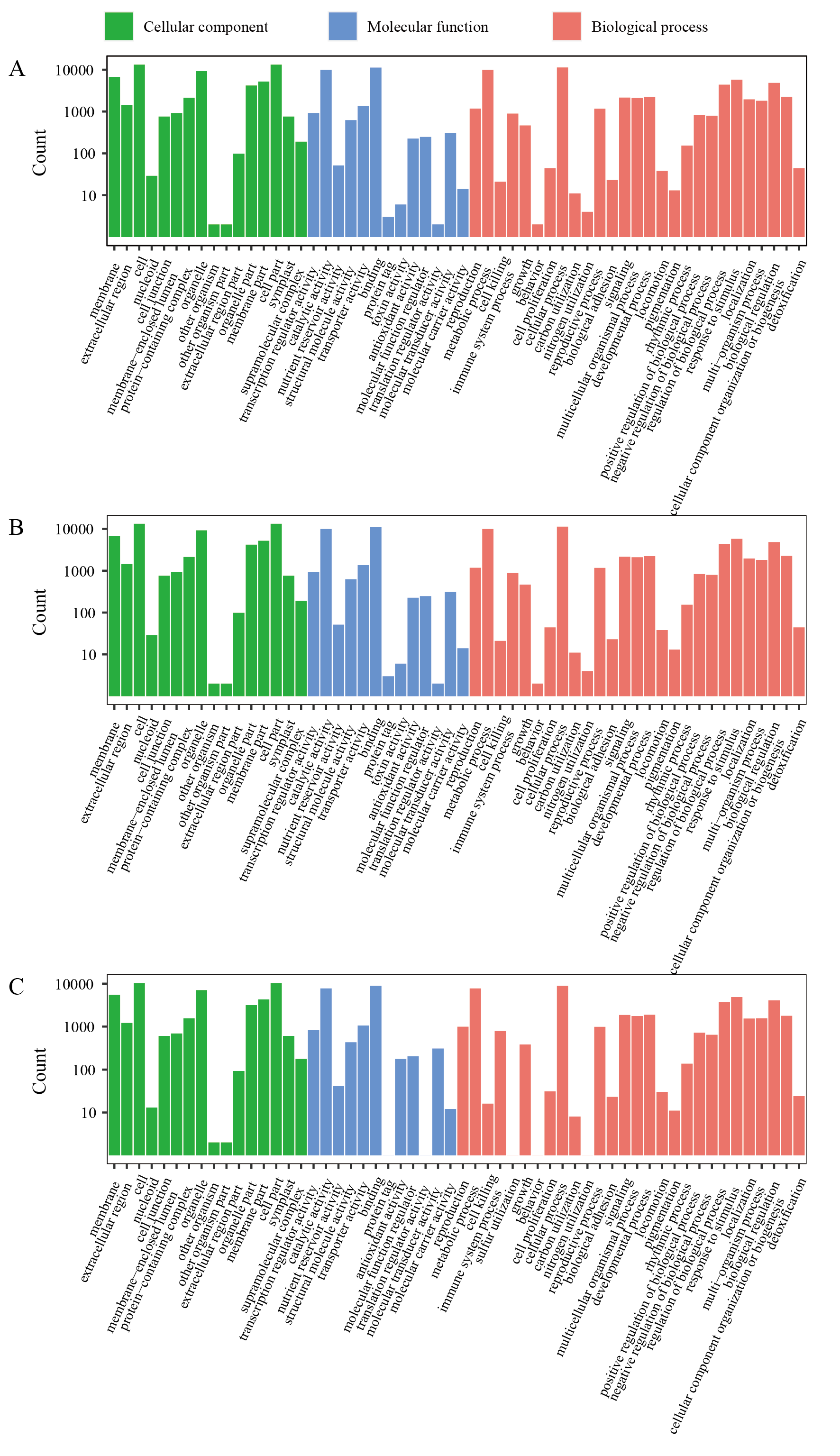

Supplement: Figure S4 — (A) GO annotation of DEGs of RM1 vs RM2. (B) GO annotation of DEGs of RM1 vs RM3. (C) GO annotation of DEGs of RM2 vs RM3. [file peerj-10-14172-s004.png]

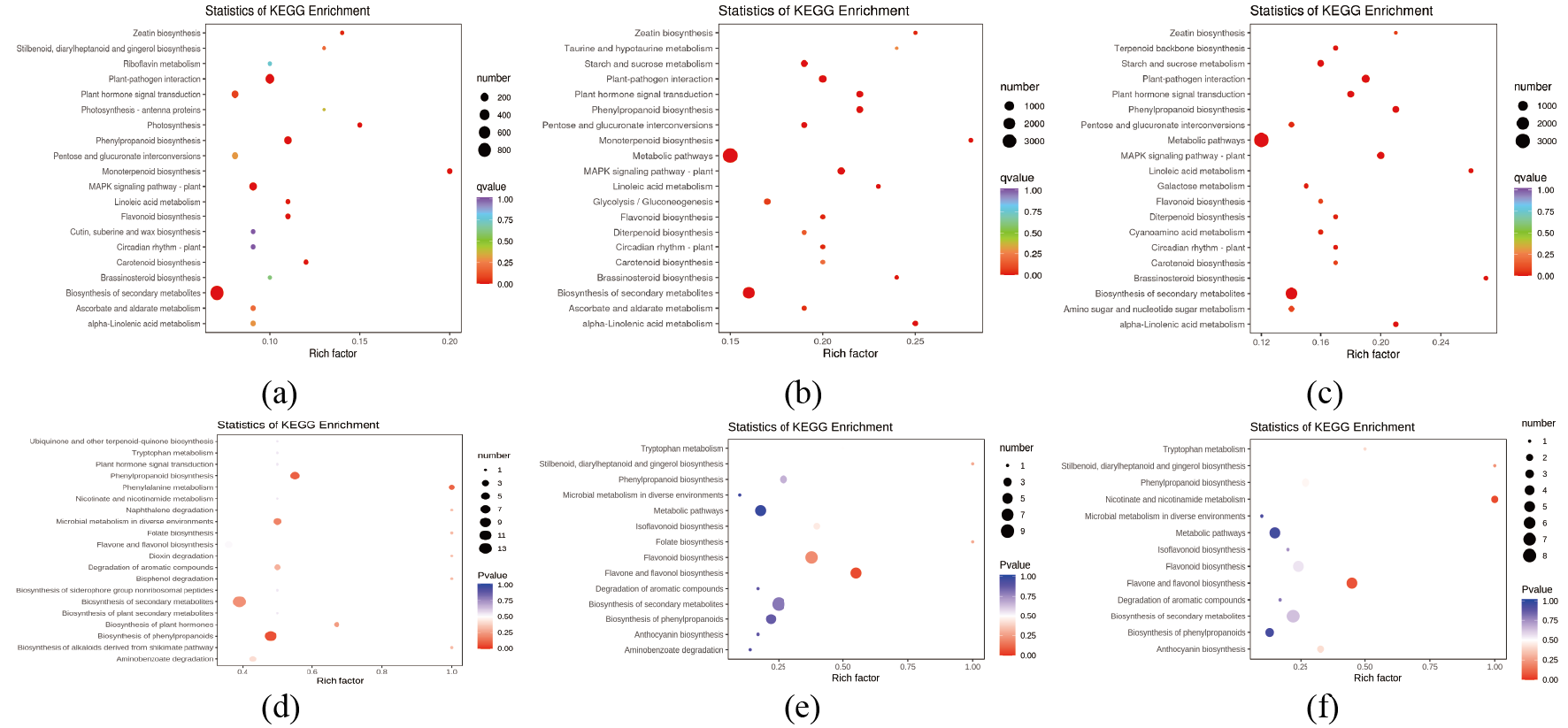

Supplement: Figure S5 — (A) KEGG enrichment of DEGs of RM1 vs RM2. (B) KEGG enrichment of DEGs of RM1 vs RM3. (C) KEGG enrichment of DEGs of RM2 vs RM3. (D) KEGG enrichment of different metabolites (DMs) of RM1 vs RM2. (E) KEGG enrichment of DMs of RM1 vs RM3. (F) KEGG enrichment of DMs of RM2 vs RM3. [file peerj-10-14172-s005.png]
